# Management and outcomes of patients with Crohn’s disease with first vs multiple surgeries: results from the PRACTICROHN study

**DOI:** 10.1093/gastro/goz029

**Published:** 2019-07-19

**Authors:** Marisa Iborra, Berta Juliá, Maria Dolores Martín Arranz, Manuel Barreiro-de Acosta, Ana Gutiérrez, Valle García-Sánchez, Carlos Taxonera, Javier P Gisbert, Luis Cea-Calvo, Eugeni Domènech

**Affiliations:** 1 Gastroenterology Department, Hospital La Fe, Valencia, Spain; 2 Centro de Investigación Biomédica en Red de Enfermedades Hepáticas y Digestivas (CIBERehd), Madrid, Spain; 3 Medical Department, Merk Sharp and Dohme (MSD), Madrid, Spain; 4 Gastroenterology Unit, Hospital Universitario La Paz, IdiPAZ, Madrid, Spain; 5 Gastroenterology Unit, Complejo Hospitalario Universitario de Santiago, Santiago de Compostela, Spain; 6 Digestive Medicine Department, Hospital General de Alicante, Alicante, Spain; 7 Unit of Clinical Management of Gastrointestinal Tract, Hospital Universitario Reina Sofia, Maimonides Institute of Biomedical Research of Cordoba (IMIBIC), Cordoba University, Cordoba, Spain; 8 Gastroenterology Department, Hospital Clínico San Carlos, Madrid, Spain; 9 Instituto de Investigación del Hospital Clínico San Carlos (IdISSC), Madrid, Spain; 10 Gastroenterology Department, Hospital Universitario de La Princesa, Instituto de Investigación Sanitaria Princesa (IIS-IP), Madrid, Spain; 11 Gastroenterology Department, Hospital Universitari Germans Trias i Pujol, Badalona, Spain

**Keywords:** Crohn’s disease, surgery, post-operative recurrence

## Abstract

**Background:**

Surgery in Crohn’s disease (CD) may be associated with poor prognosis and clinical and surgical recurrence. The aim of this study was to describe and compare the post-operative management and outcomes of patients with CD who underwent first vs recurrent surgeries.

**Methods:**

Observational study that included adult CD patients from 26 Spanish hospitals who underwent ileocolonic resection with ileocolonic anastomosis between January 2007 and December 2010. Data were retrospectively collected from the medical records.

**Results:**

Data from 314 patients were analysed, of whom 262 (83%) underwent first surgery and 52 (17%) referred to previous CD surgeries. Baseline characteristics were similar between the two groups except for a higher rate of stricturing behavior at diagnosis among re-operated patients (*P* = 0.03). After surgery, a higher proportion of re-operated patients received prophylactic treatment with immunomodulators compared with patients with first surgery (*P* = 0.04). In re-operated patients, time to clinical recurrence was not associated with the fact of receiving or not prophylaxis, whereas, in patients with first surgery, recurrence-free survival was greater when prophylaxis was received (*P* = 0.03).

**Conclusions:**

After surgery, a higher proportion of patients with previous surgeries received prophylactic treatment with immunomodulators compared with patients with first surgery. Although prophylactic treatment was beneficial for preventing clinical recurrence in patients operated on for the first time, it did not significantly reduce the risk of further recurrence in patients with previous surgeries. This suggests that effective prophylactic therapies are still needed in this subset of patients.

## Background

Approximately 50% of patients with Crohn’s disease (CD) undergo an intestinal resection within 10 years of diagnosis. Moreover, about one-fourth of operated patients will undergo a second surgery within 5 years after the first one [1]. Failure of medical treatment, stricture, fistula and abscess are the most common indications for surgery and ileocecal resection is the most prevalent type of intervention.

CD frequently recurs after an intestinal resection, although the reported rates of post-operative recurrence vary, depending on the definition (clinical, endoscopic, radiological or surgical) [2]. The reported endoscopic recurrence rates at 1 and 3 years post surgery are 73% and 85%, respectively [3]. The risk of clinical recurrence is estimated to be 20%–25% per year [4]. Previous studies have reported that new resections were required in 15%–45% of patients at 3 years, in 26%–65% at 10 years and in 33%–82% at 15 years [5].

The high incidence of post-operative recurrence in CD mandates a strict follow-up using clinical, biological and imaging techniques. Several studies tried to identify predictors for post-operative recurrence. Smoking [6], penetrating disease [7], perianal disease [8, 9] and extensive (>50-cm) resection [10] were associated with higher rates of post-operative recurrence, whereas prophylactic treatment diminished the risk of recurrence [11]. Recently, several retrospective studies have shown that anti-TNF therapy and immunomodulatory therapy, alone or in combination, seem to be an effective strategy for the prevention of CD recurrence [12–17].

Limited evidence suggests that multiple CD-related surgeries may also be a risk factor for worse outcomes and clinical recurrence at 5 years [18]. The aim of the present study was to compare the clinical management in real life and the outcomes of CD patients with only one CD-related intestinal resection (first surgery group; FSG) with patients who had more than one surgery (previous surgery group; PSG).

## Methods

### Study design and patient selection

PRACTICROHN was a retrospective study performed in 26 inflammatory bowel disease (IBD) units from Spain that aimed to explore the outcomes of patients with CD after ileocolonic surgical resection with ileocolic anastomosis over a period of 3 years–5 years [19]. Patients’ clinical records were the main source of information; no prospective data collection was allowed. The study was approved by the corresponding Ethics Research Committees. In this analysis, we describe and compare the clinical characteristics, management and outcomes of patients with CD who underwent their first CD-related surgery vs those who had referred previous surgeries.

Consecutive patients from IBD outpatient clinics aged ≥18 years who underwent CD-related ileocolonic resection with ileocolonic anastomosis between January 2007 and December 2010 were identified. All participants signed an informed consent authorizing the use of their clinical data for research purposes. For the present analysis, patients’ data from CD diagnosis and up to 5 years after surgery were retrieved from the medical records.

### Study variables

The collected data included demographics (age at CD diagnosis and surgery, gender, smoking status), clinical data (Montreal classification at diagnosis and at surgery, indication for surgery), treatments received before and after surgery, length of resection, length of hospitalization, post-operative complications, presence of residual disease after surgery, time to follow-up, prophylaxis for post-operative recurrence and colonoscopy performed within the first year after surgery.

Data regarding prophylaxis, deaths, clinical symptoms of recurrence, endoscopic results, imaging techniques (computed tomography [CT] and magnetic resonance imaging [MRI]), hospitalizations and re-interventions were collected yearly until 5 years after surgery. In case of multiple surgeries, the surgery closest to December 2010 was considered the index surgery. Post-operative clinical recurrence was defined as suggestive clinical symptoms (diarrhea, abdominal pain) and at least one of the following: Rutgeert’s endoscopic score ≥i2 and/or confirmation of disease activity by CT or MRI performed within 6 months of symptoms. Both endoscopy and imaging techniques were performed according to routine clinical practice and the investigator’s judgement. Post-operative complications comprised events recorded until 30 days after surgery and included death, ileus, anastomotic leak, digestive bleeding, abscess, wound infection, catheter-related infection and other extra abdominal infections. Prophylactic treatment for clinical post-operative recurrence was recorded at discharge or at the first outpatient visit.

### Statistical analysis

Sample size was calculated based on the objective to estimate the rate of endoscopic recurrence at 52 weeks after surgery, which had been reported to range between 36% and 56% in previous studies [15]. For an expected rate of endoscopic recurrence of 50%, it was necessary to include 267 patients to estimate the proportion of patients with disease recurrence with a confidence level of 95% and an accuracy of 6%. The sample size was increased by 15% (up to 314 individuals) to compensate for patients excluded from the analysis due to incomplete data or other reasons.

Student’s *t*-test, ANOVA or nonparametric tests (Mann–Whitney *U* test) were used to compare continuous variables, while categorical variables were compared using chi-square test or Fisher’s exact test. Probability and relative risk were assessed along with the 95% confidence interval using an exact method. Missing values and their frequency were tabulated but not included in the calculation of percentages. The distribution of the variables according to theoretical models was verified using the Kolmogorov–Smirnov test, and the assumption of homogeneity of variance was assessed using the Levene test. Post-operative clinical recurrence-free survival was assessed by means of Kaplan–Meier curves and log-rank tests. Factors associated with clinical post-operative recurrence were studied using Cox regression analysis. Variables with a *P*-value <0.15 in the univariate analysis were selected for the multivariate analysis. The null hypothesis was rejected when *P* < 0.05.

## Results

### Patients’ demographic and clinical characteristics

A total of 314 patients were included in the analysis, 52 (17%) of whom had previous surgeries before the index surgery (50 patients—one previous surgery, one patient—two surgeries and one patient—three surgeries). Table 1 summarizes patients’ demographic and clinical characteristics. Although most demographic and clinical characteristics were similar in both study groups, some differences were observed. Patients in the PSG were older and had a higher proportion of stricturing disease behavior at diagnosis, leading to a higher proportion of resection because of intestinal stenosis. More patients in the PSG had their first surgery in the first 6 months after diagnosis (37% vs 24%, *P* = 0.04). As expected, the mean disease duration at the time of index surgery was longer in the PSG than that in the FSG (12 years vs 6 years, *P* < 0.001), reflecting a more evolved disease.

**Table 1.** Demographic and clinical characteristics of patients with Crohn’s disease

Characteristic	Total (*n* = 314)	First surgery (*n* = 262)	Previous surgery (*n* = 52)	*P*-value
Male	151 (48)	132 (50)	19 (37)	0.09
Age at diagnosis, years	28.5 (2–41)	29 (22–42)	27 (22–36)	0.39
≤16 years	20 (6)	15 (6)	5 (10)	0.36
17–40 years	212 (68)	176 (67)	36 (71)	
>40 years	80 (26)	70 (27)	10 (20)	
Age at index surgery, years	39 (30–48)	37 (29–48)	41 (34–51)	0.02
Smoking status at surgery				0.48
Smoker	117 (40)	101 (41)	16 (34)	
Ex-smoker	63 (21)	50 (20)	13 (28)	
Never smoker	114 (39)	96 (39)	18 (38)	
Localization at diagnosis				0.70
L1 (± L4)	183 (59)	154 (59)	29 (57)	
L2 (± L4)	13 (4)	10 (4)	3 (6)	
L3 (± L4)	116 (37)	97 (37)	19 (37)	
Behavior at diagnosis				0.03
B1 (± P)	137 (46)	124 (49)	13 (28)	
B2 (± P)	95 (32)	74 (29)	21 (46)	
B3 (± P)	69 (23)	57 (22)	12 (26)	
Indication for surgery				0.09
Penetrating	98 (32)	84 (33)	14 (28)	
Stricturing	147 (48)	117 (46)	30 (60)	
Penetrating + stricturing	46 (15)	43 (17)	3 (6)	
Resistance to treatment	14 (5)	11 (4)	3 (6)	
Time from diagnosis to index surgery, years	–	6 ± 7	12 ± 8	<0.001
First surgery within 6 months from diagnosis	81 (26)	62 (24)	19 (37)	0.04

Values are presented as mean ± standard deviation, median (range) or *N* (%).

Missing values were not included in the calculation of percentages.

Location: L1, ileal; L2, colonic; L3, ileocolonic; and L4, isolated upper disease.

Behavior: B1, non-stricturing, non-penetrating; B2, stricturing; B3, penetrating; P, perianal disease modifier.

### Clinical management before surgery and post-operative complications

Regarding pre-operative treatment, more patients in the PSG had been exposed to corticosteroids at any time before the index surgery than in the FSG (*P* = 0.03), although, at the time of surgery, the concurrent use of corticosteroids was similar between the two groups (Table 2). Almost 40% of patients were ever exposed to immunomodulators and one-fourth to anti-TNF agents at the time of the index surgery, as well as at the time of surgery, with no differences observed between the study groups.

**Table 2.** Clinical management before and at surgery and post-operative complications

Characteristic	Total (*n* = 314)	First surgery (*n* = 262)	Previous surgery (*n* = 52)	*P*-value
Treatment before surgery, *n* (%)				
Steroids	46 (19)	33 (13)	13 (27)	0.03
Immunomodulators	114 (38)	93 (37)	21 (46)	0.34
Biologics	75 (24)	61 (24)	14 (28)	0.64
Treatment received at surgery, *n* (%)				
Steroids	81 (25)	69 (27)	12 (23)	0.70
Immunomodulators	139 (44)	112 (43)	27 (52)	0.30
Biologics	11 (21)	27 (10)	11 (21)	0.60
Length of hospitalization, median (interquartile range), days	9 (7–14)	10 (7–14)	9 (8–14)	0.32
Post-operative complications, *n* (%)	82 (26)	68 (26)	14 (27)	>0.99
Death	0 (0)	0 (0)	0 (0)	–
Ileus	10 (12)	8 (12)	2 (14)	0.68
Anastomotic leak	22 (27)	18 (26)	4 (29)	>0.99
Intra-abdominal abscess	23 (28)	20 (29)	3 (21)	0.75
Wound infection	29 (35)	24 (35)	5 (36)	>0.99
Sepsis	3 (4)	3 (4)	0 (0)	>0.99
Gastrointestinal bleeding	9 (11)	9 (13)	0 (0)	0.35
Extra-intestinal infections	10 (12)	6 (9)	4 (29)	0.06

Missing values were not included in the calculation of percentages.

A total of 82 (26%) patients experienced post-operative complications, most frequently wound infections, extra-intestinal infections or anastomotic leak, but no significant differences between the two groups were found regarding the rate of post-operative complications or length of hospital stay (Table 2).

### Clinical management after surgery

After surgery, a total of 208 (68%) patients received prophylactic treatment for the prevention of post-operative recurrence. Prophylaxis was prescribed similarly to patients with and without previous surgeries (66% of patients in the FSG and 76% of patients in the PSG, *P* = 0.20; Table 3). However, a higher proportion of patients in the PSG received prophylactic treatment with immunomodulators compared to the FSG (59% vs 43%, *P* = 0.04), whereas antibiotics were used less and only in patients with first surgery (0% vs 11%, *P* = 0.03).

**Table 3.** Clinical management after surgery

Characteristic	Total (*n* = 314)	First surgery (*n* = 262)	Previous surgery (*n* = 52)	*P*-value
Time to follow-up visit, median (interquartile range), days	33 (14–55)	32 (16–53)	38 (20–68)	0.31
Post-operative prophylaxis received, *n* (%)	208 (68)	169 (66)	39 (76)	0.19
Post-operative prophylaxis,^a^*n* (%)				
Immunomodulators	141 (46)	111 (43)	30 (59)	0.04
Antibiotics	27 (9)	27 (11)	0 (0)	0.03
Aminosalycilates	39 (13)	30 (12)	9 (18)	0.25
None	99 (32)	87 (34)	12 (23)	–
Colonoscopy performed within the first year, *n* (%)	181 (58)	154 (59)	27 (52)	0.39
Residual disease after surgery	22 (7)	18 (7)	4 (8)	0.77
Post-operative prophylaxis in patients with residual disease,^a^*n* (%)				
Immunomodulators	8 (36)	6 (33)	2 (50)	0.60
Antibiotics	6 (27)	6 (33)	0 (0)	0.54
Aminosalycilates	8 (36)	6 (33)	2 (50)	0.60
None	6 (27)	5 (27)	1 (25)	–
Colonoscopy scheduled in the first year in patient with residual disease, *n* (%)	12 (54)	10 (56)	2 (50)	>0.99

^a^ Recorded at discharge or at the first control visit.

Missing values were not included in the calculation of percentages.

Time to first control visit and rates of colonoscopies performed during the first year after surgery were similar between the two cohorts. A similar proportion of patients had residual disease after the surgery in both cohorts; the post-operative management of these patients was similar to that of the other patients (Table 3).

### Outcomes after surgery

**Figure 1.**
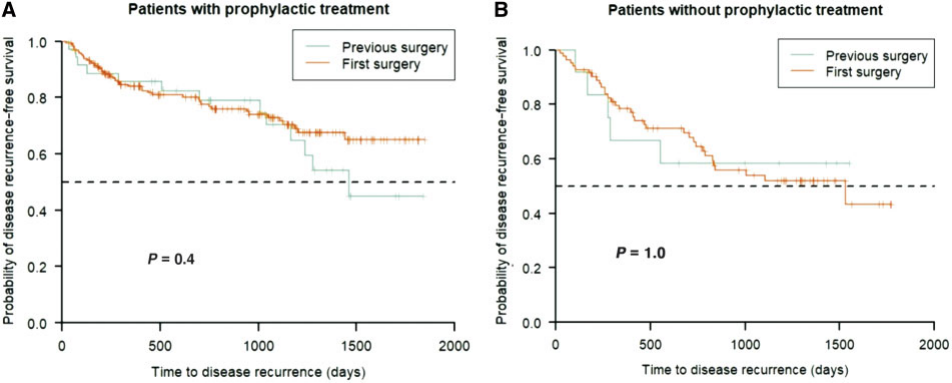
Kaplan–Meyer curves for time to clinical recurrence in (A) patients who received prophylactic treatment and (B) patients who did not receive prophylactic treatment

Within the first year of follow-up, 11 (23%) patients in the PSG and 62 (24%) patients in the FSG experienced clinical recurrence (Table 4). When evaluating the effect of previous surgeries on clinical recurrence-free survival in patients receiving or not prophylaxis, we found no statistical differences (Figure 1).

**Table 4.** Outcomes after surgery

Characteristic	Total (*n* = 314)	First surgery (*n* = 262)	Previous surgery (*n* = 52)	*P*-value
Post-operative clinical recurrence within the first year, *n* (%)	73 (23)	62 (24)	11 (21)	0.8
Rutgeert’s endoscopic score at 5 years after surgery, *n* (%)				0.85
i0–i1	116 (46)	100 (46)	16 (43)	
≥i2	136 (54)	115 (53)	21 (57)	
Hospitalizations within 5 years after surgery, *n* (%)	94 (30)	74 (28)	20 (39)	0.17
Re-interventions within 5 years after surgery, *n* (%)	45 (15)	38 (15)	7 (14)	>0.99
Hospitalizations within 5 years after surgery in patients with residual disease, *n* (%)	15 (68)	12 (67)	3 (75)	>0.99
Re-interventions within 5 years after surgery in patients with residual disease, *n* (%)	6 (27)	3 (17)	3 (75)	0.05

**Figure 2.**
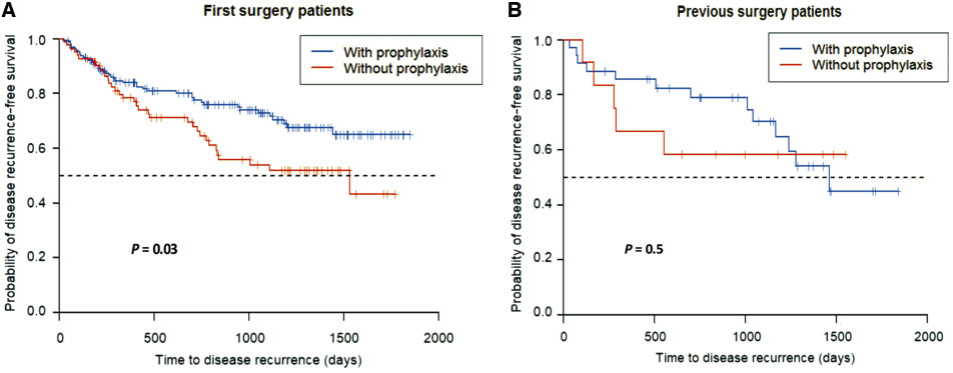
Kaplan–Meyer curves for time to clinical recurrence in (A) patients who underwent first surgery and (B) patients who underwent repetitive surgeries

We also analysed clinical recurrence-free survival according to the administration of the prophylaxis for post-operative recurrence. In the FSG, clinical recurrence-free survival time was longer in patients with prophylactic treatment (*P* = 0.03). However, there was no significant difference in clinical recurrence-free survival between patients with and without prophylaxis in the PSG (*P* = 0.5; Figure 2).

The multivariate Cox regression analysis identified that receiving no prophylactic treatment or only antibiotic treatment, as well as the time from diagnosis to surgery, were independent predictors of post-operative recurrence. Conversely, no residual disease was associated with less post-operative recurrence (Table 5).

**Table 5.** Prognostic factors associated with post-operative recurrence using multivariate Cox regression analysis

Variable	Coeficient	e^coeficient^	*P*-value
Sex: woman	–0.194	0.824	0.540
Age at the time of surgery	0.005	1.005	0.760
Disease behavior at the time of surgery: B2 ± P	–0.704	0.494	0.220
Disease behavior at the time of surgery: B3 ± P	–1.007	0.365	0.098
Prophylaxis: antibiotics	1.054	2.870	0.035
Prophylaxis: aminosalicylates	0.580	1.786	0.240
Prophylaxis: without prophylaxis	0.753	2.122	0.043
Residual disease: no	–1.004	0.366	0.019
Time between diagnosis and first surgery	0.046	1.047	0.029
Smoker at the time of surgery	–0.146	0.864	0.710

Post-operative endoscopic recurrence (Rutgeert’s score ≥i2) was observed in 54% at 5 years’ follow-up of all patients, with similar percentages in both cohorts. A total of 94 (30%) patients required hospitalization and 45 (15%) required re-intervention in the 5 years of follow-up without significant differences between the PSG and the FSG (Table 4).

## Discussion

In this study, we analysed the management and treatment outcomes of 314 patients with CD who underwent ileocolonic resection with ileocolonic anastomosis, 52 of whom had been previously operated on. We found no differences in clinical management between the PSG and the FSG except for the prophylaxis with immunomodulators, which were more frequently administered in the PSG. However, prophylactic therapy only resulted in clinical benefit in patients in the FSG, showing a reduction in the rate of post-operative clinical recurrence at 5 years, which was not observed in the PSG.

Similarly, there were no significant differences in the rates of endoscopic, clinical or surgical recurrences between patients operated on for the first time and those who had previous surgeries; whether the lack of differences could be due to more prophylactic treatment received by the PSG mentioned above would be a matter of debate. Our findings agree with a previous report by Heimann *et al*. [18], who found that the rates of post-operative clinical recurrence at 3 years after first and second (or more) operations were similar (41% and 39%, respectively), although the relapse rate increased significantly in patients with three or more previous resections. The authors concluded that a disease requiring multiple re-operations is more aggressive and is more likely to result in complex resections, perioperative blood transfusions and permanent ileostomy [18]. In our study, there were very few patients with more than one re-operation, so we were unable to corroborate these conclusions.

In our study, no differences in endoscopic procedures during the first year after the index surgery were found between the FSG and the PSG, probably reflecting the time at which the study was performed (2007–2010). Accordingly, a previous publication of the results of the PRACTICROHN study demonstrated a trend towards an increased rate of endoscopies performed during the first year after surgery from 2007 to 2010 [19].

Clinical guidelines suggest the use of prophylactic treatment after ileocolonic intestinal resection in patients with at least one risk factor for recurrence (evidence level 2). The drugs of choice to prevent post-operative recurrence are thiopurines (evidence level 2) and anti-TNFs (evidence level 2). In our study, even though most patients received prophylactic treatment (68%), the use of prophylaxis in general and of immunomodulators in particular was more frequent in re-operated patients. This may reflect the urge for better prevention felt by the physicians in case of re-operated patients, since previous surgeries have been suggested to be a risk factor of poor outcomes in CD patients and have been associated with poor prognosis [20]. Additionally, as reported in the previous publication of the PRACTICROHN study [19], only three patients received prophylaxis with anti-TNF and only one patient on monotherapy, so we are not able to analyse the effect of anti-TNF prophylaxis on clinical outcomes in this population. Our multivariate analysis also showed that not receiving prophylaxis is a risk factor for post-operative clinical recurrence. In the meta-analysis by Frolkis *et al.* [1], around one out of four patients with CD underwent a second surgery within 5 years of their first resection, but later the risk of a second surgery increased more slowly (from 25% at 5 years to 35% at 10 years), hence strategies to prevent re-interventions in the first 5 years after surgery may result in better disease outcomes. Our data showed that 45 (15%) patients required re-intervention over the 5 years’ follow-up. The lack of difference in the rates of re-interventions between the FSG and the PSG observed in our study could be due to the insufficient size of the PSG, which could be too small to detect any differences, as well as too short a follow-up period.

A small (7%–8%) and similar proportion of patients in the PSG and the FSG had residual disease after resection. The multivariate analysis showed that residual disease was associated with higher risk of post-operative clinical recurrence. Interestingly, the management of these patients (prophylactic treatment, scheduled colonoscopies) did not differ from the management of the rest. However, the long-term outcomes among patients with residual disease were substantially worse: the rates of hospitalizations and re-interventions doubled those of the total patient population. This observation held true both for patients with first surgery and for those with previous surgeries. Besides, the rate of re-interventions was significantly higher in re-operated patients with residual disease, compared to those with first surgery and residual disease. However, the small number of patients and borderline level of significance preclude drawing strong conclusions. Our findings suggest that more research on management and outcomes in these patients should be done and that specific management algorithms may be needed for this patient population.

As the time from diagnosis to the first surgery is associated with a higher probability of recurrence, this would imply that, in some patients, an early surgery would be a good strategy to improve long-term disease management, as shown in the study by An *et al.* [21], which suggests that patients with ileocolonic CD may have a more benign disease course if undergoing early surgical intervention, with fewer admissions to hospital and a trend towards reduced overall operation rates, although it would be necessary to design studies that explore this aspect in depth.

Our study has several limitations to consider. First, because of the study’s retrospective design, the collected data were limited to those available from patients’ medical records. However, the available data from clinical records were quite complete and allowed drawing conclusions. Second, this was not a randomized study, and therefore the decision to give a prophylactic treatment for recurrence was not random, but a physician’s decision according to the patient’s risk of recurrence. Similarly, since this was an observational study, colonoscopy was not done systematically in all patients, but according to routine clinical practice and the physician’s decision. Another limitation is that, although the overall sample size was quite large (314 patients), only 52 (17%) patients referred previous surgeries. Thus, some differences in characteristics, management or outcomes may have stayed undetected because of insufficient statistical power. On the other hand, among the strengths of the study, we could indicate a long (5 years’) follow-up and its real-life, multicenter character that allows careful extrapolation onto routine management of this challenging condition.

In conclusion, in our study, patients with CD who had previous surgeries were more frequently treated with prophylactic immunomodulators but had a similar rate of endoscopic, clinical or surgical CD recurrence compared with patients operated on for the first time. Although prophylactic treatment was beneficial for preventing clinical recurrence in patients operated on for the first time, it did not significantly reduce the risk of further recurrence in patients with previous surgeries. This suggests that a history of a previous surgery may be a poor prognostic factor in patients with CD, and new strategies to prevent recurrence in these patients may be needed.

## Authors’ contribution

Study concept and protocol development: E.D., M.I., B.J., L.C.C., M.B.A., A.G., V.G.S and D.M.A. Manuscript writing: M.I., B.J. and E.D. All authors read and approved the final manuscript.

## Funding

This study was funded by Merck Sharp & Dohme of Spain, a subsidiary of Merck & Co., Inc., Kenilworth, New Jersey, USA.
